# Trajectories of perinatal depression, anxiety, perceived stress, sleep quality, and psychological flexibility from pregnancy to six months postpartum and their relationship with the number of abortions and deliveries

**DOI:** 10.3389/fpsyt.2026.1973469

**Published:** 2026-10-28

**Authors:** Yan Gao, Jingwen Sun, Lei Yang, Yongai Zhang, Yiping Nan, Xiaomei Li

**Affiliations:** 1 School of Nursing, Health Science Centre, Xi’an Jiaotong University, Xi’an, China; 2 Nursing Department, The Second Affiliated Hospital of Xi’an Medical University, Xi’an, China; 3 The School of Nursing and Rehabilitation, Xi’an Jiaotong University City College, Xi’an, China; 4 Nursing and Rehabilitation College, Xi’an Medical University, Xi’an, China

**Keywords:** abortion, anxiety, group-based trajectory modelling, perceived stress, perinatal depression, psychological flexibility, sleep quality

## Abstract

**Background:**

Perinatal psychological distress is common and longitudinally heterogeneous, yet little is known about how obstetric history shapes symptom trajectories. We identified trajectories of depressive symptoms, perceived stress, sleep quality, psychological flexibility, and anxiety from early pregnancy to 24 weeks postpartum and examined their associations with the number of previous abortions and deliveries.

**Methods:**

In this prospective longitudinal cohort study, 254 pregnant women were recruited at 13 weeks of gestation from a tertiary hospital in China and assessed eight times through 24 weeks postpartum using the EPDS, CPSS, PSQI, CompACT, and GAD-7. Group-based trajectory modeling identified latent trajectory classes. Logistic regression examined associations with obstetric history, adjusting for sociodemographic covariates.

**Results:**

Three trajectories emerged for depressive symptoms (low-stable, 38.98%; mild-stable, 47.24%; moderate-increasing, 13.78%), perceived stress (low-stable, 31.89%; moderate-increasing, 16.54%; high-stable, 51.57%), and sleep quality (good-stable, 50.00%; mild-worsening, 38.58%; persistent poor, 11.42%); two emerged for psychological flexibility (continuously declining vs high-stable) and anxiety (low-stable, 93.70%; moderate, 6.30%). Compared with women without abortion history, those with two or more abortions had higher odds of mild-stable (OR = 5.14) and moderate-increasing depression (OR = 19.12), mild-worsening (OR = 3.06) and persistent poor sleep (OR = 5.51), and declining psychological flexibility (OR = 2.61); one abortion was associated with increased stress (OR = 2.54 and 2.16) and moderate anxiety (OR = 4.18). Number of deliveries was not associated with any trajectory.

**Conclusions:**

Previous abortions predict adverse perinatal psychological trajectories in a dose-dependent manner. Routine assessment of abortion history at the first prenatal visit could help target early screening and tailored interventions.

## 1 Introduction

Maternal mental health during the perinatal period is a critical determinant of both maternal well-being and offspring development (1, 2). Perinatal depression, anxiety, and stress are highly prevalent worldwide, with pooled estimates suggesting that approximately 16.3% of women in mainland China experience clinically significant depressive symptoms during the perinatal period, and that antenatal depression (19.7%) is even more common than postnatal depression (14.8%) (3). Beyond depression, anxiety symptoms, poor sleep quality, and elevated perceived stress frequently co-occur during this window, and are independently associated with adverse obstetric outcomes, impaired mother–infant bonding, and long-term child neurodevelopmental deficits (4–6). Despite this substantial burden, perinatal psychological distress remains under-recognized and under-treated in routine obstetric care (3, 7).

Longitudinal studies have shown that perinatal psychological symptoms exhibit significant interindividual heterogeneity over time, with three to five distinct trajectories of depressive symptoms identified, including stable low levels, progressive increase, progressive decrease, and persistently high levels (7–10). Similar heterogeneity has been observed for anxiety and sleep quality (5, 11, 12). This longitudinal analytical approach has revealed clinically significant subgroups that were previously masked (7). Nevertheless, most studies focus on a single outcome, typically depression or anxiety. Second, although demographic and psychosocial predictors such as social support and pregnancy-related anxiety have been studied, little is currently known about the role of obstetric history, particularly the number of previous miscarriages and live births, as a factor influencing different psychological trajectories.

Prior pregnancy loss is a common reproductive experience, affecting approximately 20% of confirmed deliveries, and is considered a persistent source of psychological stress (13, 14). Compared with women without a history of miscarriage, those with prior miscarriage report significantly higher levels of pregnancy-specific anxiety, depressive symptoms, and perceived stress in subsequent deliveries (15–17). Importantly, a growing body of evidence suggests a dose-response relationship: women who have experienced two or more miscarriages report higher levels of anxiety and depressive symptoms than those who have experienced only one (16, 18–20). In contrast, the number of deliveries appears to have a more complex and potentially bidirectional effect (21, 22). Moreover, psychological flexibility has been shown to be a protective factor for perinatal mental health (6). Lower psychological flexibility is associated with higher perinatal anxiety and depression, while Acceptance and Commitment Therapy (ACT) targeting psychological flexibility has been shown to be effective in alleviating perinatal psychological distress (6, 23). However, whether psychological flexibility follows distinct developmental trajectories during the perinatal period, and whether these trajectories are influenced by reproductive history, has not yet been systematically investigated.

This study employs a prospective longitudinal design, involving eight repeated assessments conducted between the 13th week of pregnancy and the 24th week postpartum. By simultaneously modeling the trajectories of five psychological Outcomes: depressive symptoms, anxiety, perceived stress, sleep quality and psychological flexibility, the study will investigate the association between the number of previous miscarriages and deliveries at baseline and psychological trajectories during pregnancy. The findings are expected to provide a basis for developing risk-stratified screening programmers and personalized psychological interventions tailored to women with different reproductive histories, thereby advancing the precision of perinatal mental health care.

## 2 Materials and methods

### 2.1 Study design and participants

This prospective longitudinal cohort study was conducted in the obstetric outpatient department of a tertiary hospital in China. Pregnant women were consecutively recruited at their first prenatal visit between March and May 2022. Eligible participants were women aged 20 years or older, confirmed as early pregnancy by ultrasound, able to read and understand Chinese, and willing to complete the follow-up assessments. Women were excluded if they had a diagnosed severe psychiatric disorder (e.g., schizophrenia, bipolar disorder) or current use of psychotropic medication, had severe fetal abnormalities requiring termination of pregnancy, or had cognitive impairment that would preclude questionnaire completion.

The sample size was estimated using the formula for a quantitative indicator in a prevalence study with antenatal anxiety as the primary calculation indicator. According to a previous meta-analysis, the prevalence of anxiety among pregnant women was taken as *π* = 17.4% (24). To ensure precision, the allowable error was set at *δ* = 0.05, and the 95% confidence level was adopted. Accounting for an anticipated 20% attrition rate across the eight repeated assessments, the target sample size was inflated to 264 participants. Of the 298 enrolled women, 44 who completed fewer than three follow-up assessments were excluded; baseline characteristics did not differ between included and excluded participants (all P > 0.05; **Supplementary Table S1**).

The study protocol was approved by the Ethics Committee of Xi’an Jiaotong University (approval number: 2022-1436). Written informed consent was obtained from all participants prior to enrollment. All methods were performed in accordance with the Declaration of Helsinki and relevant guidelines.

### 2.2 Data collection and measurement time points

Study participants completed interview questionnaires at eight time points: at 13 weeks of gestation (baseline), 20, 26, 32, and 37 weeks, as well as at 1, 6, and 24 weeks postpartum. At baseline, professionally trained research nurses collected demographic and obstetric information, including age, educational level, residence, occupation, average monthly household income, family history of depression, number of previous deliveries, and number of previous abortions (including spontaneous and induced). The number of deliveries refers to the number of times a pregnant woman has given birth to a fetus at 28 weeks’ gestation or later. Abortion refers to the termination of a pregnancy before 28 weeks’ gestation, with a fetal weight of less than 1,000 grams. At each time point, a face-to-face psychological assessment was conducted using questionnaires.

### 2.3 Measures

#### 2.3.1 Depressive symptoms

Depressive symptoms were assessed using the Edinburgh Postnatal Depression Scale (EPDS), which consists of 10 items. Originally developed for the postpartum population, the validity of this scale for use during pregnancy has been widely validated (25, 26). Each item is scored on a 4-point scale (0–3), with a total score ranging from 0 to 30. A higher score indicates more severe depressive symptoms. The scale demonstrated good internal consistency in our sample data, with Cronbach’s α coefficients ranging from 0.806 to 0.863 across all time points (see **Supplementary Table S2**).

#### 2.3.2 Perceived stress

Perceived stress was assessed using the 14-item Chinese Perceived Stress Scale (CPSS) (27). Items are rated on a 5-point Likert scale (0 = never to 4 = always), with seven positively stated items (4–7, 9, 10, 13) reverse-scored. The total score ranges from 0 to 56, with higher scores indicating greater perceived stress. The scale demonstrates good internal consistency, with Cronbach’s α coefficients ranging from 0.799 to 0.878 across all time points (**Supplementary Table S2**).

#### 2.3.3 Sleep quality

Sleep quality over the preceding month was measured with the Pittsburgh Sleep Quality Index (PSQI) (28). The 19 self-rated items generate seven component scores that sum to a global score of 0–21, with higher scores indicating poorer sleep quality. The scale demonstrates good internal consistency, with Cronbach’s α coefficients ranging from 0.717 to 0.898 across all time points (**Supplementary Table S2**).

#### 2.3.4 Psychological flexibility

Psychological flexibility was measured using the 15-item short form of the Comprehensive Assessment of Acceptance and Commitment Therapy Processes (CompACT) (29). The scale comprises three dyadic subscales: Openness to Experience (items 1–3), Behavioral Awareness (items 4–8), and Valued Action (items 9–15). Items 1–3 and 8 are reverse scored, and the total score ranges from 0 to 90, with higher scores indicating greater psychological flexibility. The scale demonstrates good internal consistency, with Cronbach’s α coefficients ranging from 0.852 to 0.908 across all time points (**Supplementary Table S2**).

#### 2.3.5 Anxiety symptoms

Anxiety was measured with the 7-item Generalized Anxiety Disorder scale (GAD-7) (30). Each item is scored from 0 (not at all) to 3 (nearly every day), giving a total score of 0–21. Scores of 5–9, 10–14, and ≥15 indicate mild, moderate, and severe anxiety, respectively. The scale demonstrates good internal consistency, with Cronbach’s α coefficients ranging from 0.865 to 0.931 across all time points (**Supplementary Table S2**).

### 2.4 Statistical analysis

Descriptive statistics were used to characterize the sample. The number of abortions and deliveries at baseline was self-reported by maternal participants and categorized as 0, 1, or ≥2. Descriptions were provided for each group using frequencies and percentages for categorical variables, and Chi-square tests were used for comparisons between groups. The group-based trajectory modeling (GBTM), a semi-parametric finite mixture modeling approach, was applied to identify latent subgroups following distinct developmental courses for each of the five psychological outcomes (EPDS, CPSS, GAD-7, PSQI, and CompACT) (31, 32). GBTM accommodates intermittently missing data under maximum likelihood estimation, using all available observations. Time was coded as gestational/postpartum week. Models with one to six trajectory classes were estimated for each outcome using a censored normal distribution, with polynomial functions (intercept, linear, quadratic terms) tested for each class. The optimal number of classes was determined by: (1) lower Bayesian Information Criterion (BIC) and Akaike Information Criterion (AIC) values; (2) entropy approaching 1.0; (3) average posterior probability (avePP) of assignment ≥ 0.70 for each class; and (4) each class comprising at least 5% of the sample with clinical interpretability (32).

Following trajectory identification, associations between baseline obstetric history (number of previous abortions: 0, 1, ≥ 2; number of previous deliveries: 0, 1, ≥ 2) and trajectory class membership were examined using binary or multinomial logistic regression, reporting odds ratios (ORs) with 95% confidence intervals (CIs). Two models were fitted: Model 1 was unadjusted; Model 2 adjusted for age, education level, average monthly household income, residence, occupation, and family history of depression. All analyses were performed using R version 4.3.3. Two-sided P values <0.05 were considered statistically significant.

## 3 Results

### 3.1 Participant characteristics

The participant flow is shown in **Figure 1**. A total of 254 pregnant women were included in the trajectory analysis. Grouped by the number of previous abortions, 128 women (50.39%) reported no history of abortion, 86 (33.86%) reported one, and 40 (15.75%) reported two or more; grouped by the number of previous deliveries, 127 (50.00%) were in their first pregnancy, 99 (38.98%) had been pregnant once before, and 28 (11.02%) twice or more (**Table 1**). Women with a history of abortion were significantly less likely to hold a bachelor’s degree or above (P = 0.005); a family history of depression was also more frequent among women with prior abortions, although this difference did not reach statistical significance (P = 0.076). Compared with first-pregnancy women, women with one or more previous deliveries were significantly older (P = 0.001), less educated (P = 0.003), and exercised less frequently (P = 0.001). Notably, satisfaction with the parental, marital, and mother-in-law relationships decreased significantly with an increasing number of both previous abortions and previous deliveries. No significant group differences were observed in average monthly household income, residence, occupation, mode of conception, or pregnancy complications (all P > 0.05).

**Figure 1:**
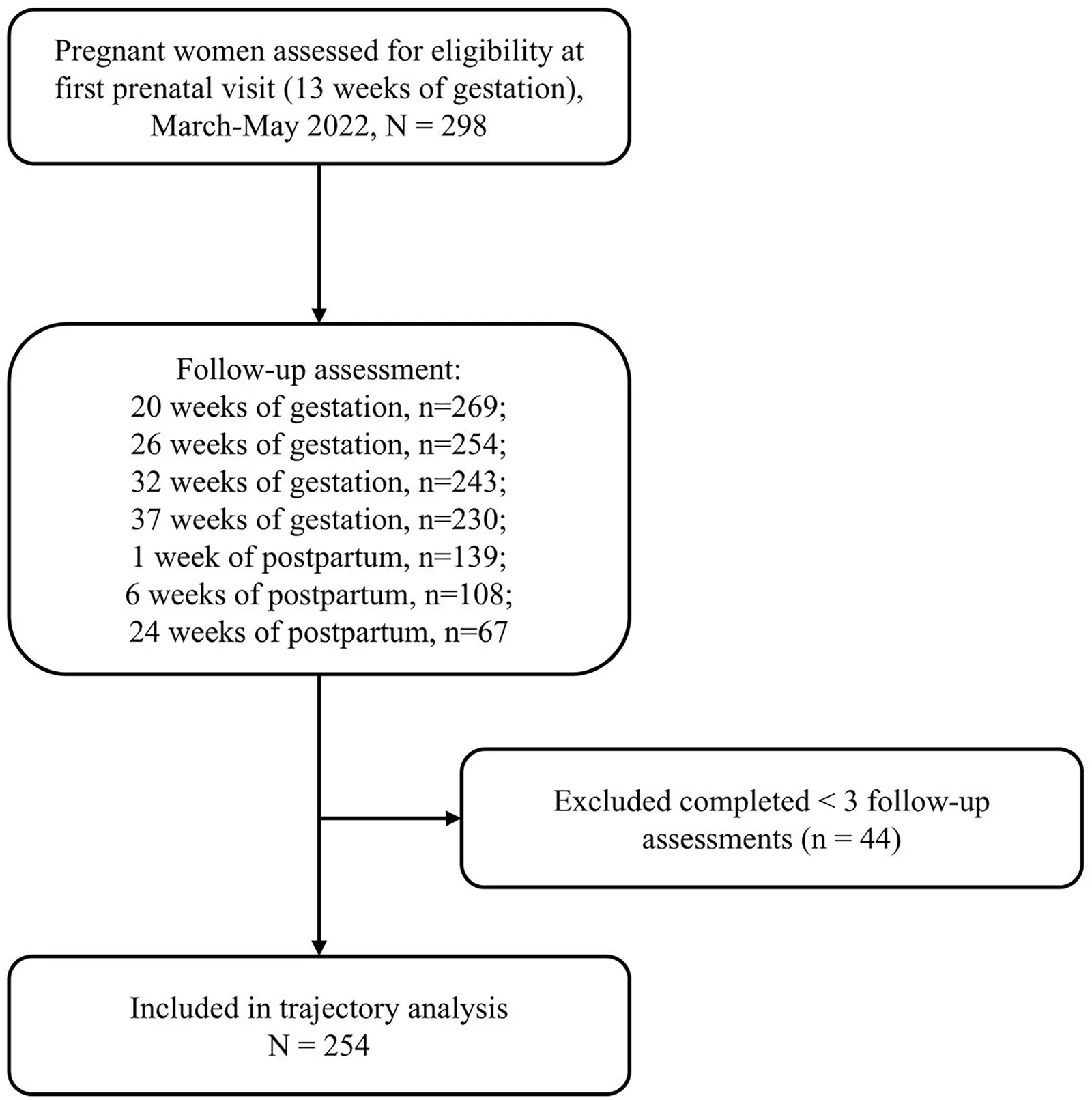
Flowchart of the study sample selection process.

**Table 1:** Baseline characteristics of participants grouped by number of abortions and deliveries.

Characteristics	The number of abortions				The number of deliveries			
	0	1	≥ 2	*P* value	0	1	≥ 2	*P* value
	N = 128	N = 86	N = 40		N = 127	N = 99	N = 28	
Age, *n* (%)								
< 30	72 (56.25)	43 (50.00)	14 (35.00)	0.063	95 (74.80)	28 (28.28)	6 (21.43)	0.001
≥ 30	56 (43.75)	43 (50.00)	26 (65.00)		32 (25.20)	71 (71.72)	22 (78.57)	
Education, *n* (%)								
High school or below	34 (26.56)	39 (45.35)	19 (47.50)	0.005	33 (25.98)	46 (46.46)	13 (46.43)	0.003
Bachelor or above	94 (73.44)	47 (54.65)	21 (52.50)		94 (74.02)	53 (53.54)	15 (53.57)	
Average monthly household income, *n* (%)								
< 6,000	84 (65.62)	57 (66.28)	28 (70.00)	0.876	82 (64.57)	68 (68.69)	19 (67.86)	0.799
≥ 6,000	44 (34.38)	29 (33.72)	12 (30.00)		45 (35.43)	31 (31.31)	9 (32.14)	
Residence, *n* (%)								
Urban	85 (66.41)	60 (69.77)	26 (65.00)	0.827	86 (67.72)	68 (68.69)	17 (60.71)	0.723
Rural	43 (33.59)	26 (30.23)	14 (35.00)		41 (32.28)	31 (31.31)	11 (39.29)	
Occupation, *n* (%)								
Unemployed	33 (25.78)	29 (33.72)	17 (42.50)	0.111	33 (25.98)	36 (36.36)	10 (35.71)	0.211
Employed	95 (74.22)	57 (66.28)	23 (57.50)		94 (74.02)	63 (63.64)	18 (64.29)	
Family history of depression, *n* (%)								
No	125 (97.66)	79 (91.86)	36 (90.00)	0.076	123 (96.85)	92 (92.93)	25 (89.29)	0.194
Yes	3 (2.34)	7 (8.14)	4 (10.00)		4 (3.15)	7 (7.07)	3 (10.71)	
Mode of conception, *n* (%)								
Natural	126 (98.44)	84 (97.67)	40 (100.00)	0.621	126 (99.21)	96 (96.97)	28 (100.00)	0.315
Assisted	2 (1.56)	2 (2.33)	0 (0.00)		1 (0.79)	3 (3.03)	0 (0.00)	
Exercise frequency / week, *n* (%)								
≥ 5 times	21 (16.41)	19 (22.09)	5 (12.50)	0.858	35 (27.56)	9 (9.09)	1 (3.57)	0.001
3–4 times	29 (22.66)	20 (23.26)	11 (27.50)		27 (21.26)	22 (22.22)	11 (39.29)	
1–2 times	30 (23.44)	20 (23.26)	9 (22.50)		29 (22.83)	22 (22.22)	8 (28.57)	
Rarely	48 (37.50)	27 (31.40)	15 (37.50)		36 (28.35)	46 (46.46)	8 (28.57)	
Complications, *n* (%)								
No	119 (92.97)	71 (82.56)	35 (87.50)	0.062	117 (92.13)	84 (84.85)	24 (85.71)	0.205
Yes	9 (7.03)	15 (17.44)	5 (12.50)		10 (7.87)	15 (15.15)	4 (14.29)	
Parental relationship satisfaction								
M (IQR)	9.50 (8.00, 10.00)	9.00 (8.00, 10.00)	9.00 (7.75, 10.00)	0.018	10.00 (8.00, 10.00)	9.00 (8.00, 10.00)	9.00 (7.00, 10.00)	0.046
Marital relationship satisfaction								
M (IQR)	9.00 (8.00, 10.00)	9.00 (8.00, 9.75)	8.50 (7.75, 10.00)	0.003	9.00 (8.00, 10.00)	9.00 (8.00, 10.00)	9.00 (7.75, 10.00)	0.016
Mother-in-law relationship satisfaction								
M (IQR)	9.00 (8.00, 10.00)	8.00 (6.25, 9.00)	8.00 (6.00, 9.25)	0.007	8.00 (7.50, 10.00)	8.00 (7.00, 9.00)	8.00 (7.00, 9.00)	0.037

Categorical variables were analyzed using the Chi−square test, and continuous variables using the Mann–Whitney U test.

**Figure 2** presents the score distributions of the five psychological measures across the eight assessment points. EPDS scores were highest and most widely dispersed at baseline (13 weeks of gestation) and declined to lower, stable levels from mid-pregnancy onward. CPSS scores remained broadly stable around moderate levels throughout. PSQI scores increased gradually across pregnancy, peaked in the early postpartum period, and declined slightly thereafter. CompACT scores were stably distributed around the mid-range. and GAD-7 scores remained low overall, with a mild elevation in late pregnancy. At every time point, the distributions were right-skewed, indicating a subgroup of women with persistently elevated symptoms.

**Figure 2:**
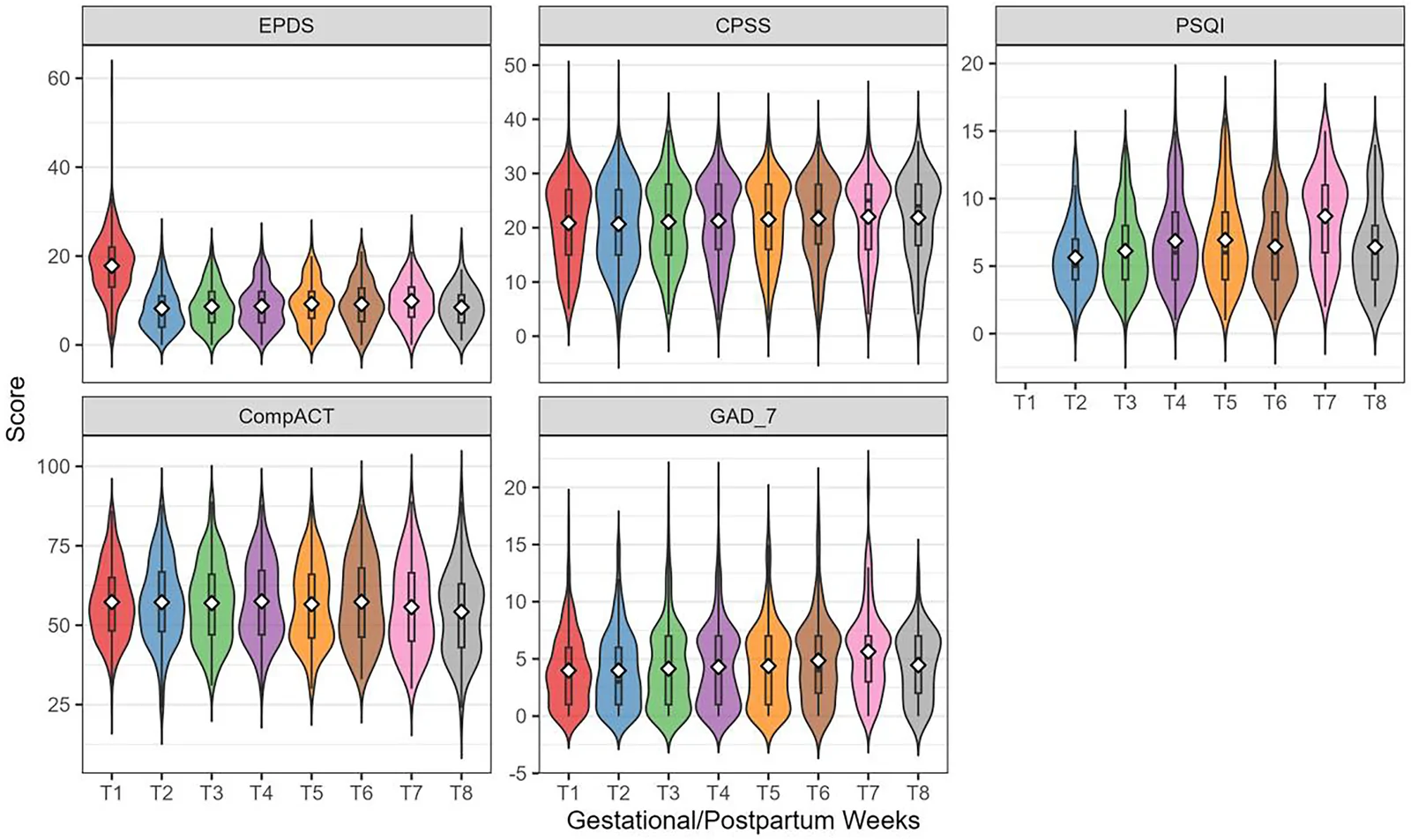
Distribution of scores for depressive symptoms (EPDS), perceived stress (CPSS), sleep quality (PSQI), psychological flexibility (CompACT), and anxiety (GAD-7) at each assessment time point.

### 3.2 Trajectories of depressive symptoms (EPDS)

Model fit indices for one- to six-class GBTM solutions are presented in **Supplementary Table S3**. The three-class model was selected as optimal, balancing fit improvement (AIC = 7715.20, BIC = 7768.26), acceptable entropy (0.74), avePP (0.85–0.92), and class sizes (all >5%). The three trajectories were: Class 1 (38.98%), characterized by persistently low depressive symptoms (low-stable); Class 2 (47.24%), with mild symptoms that remained relatively flat across the perinatal period (mild-stable); and Class 3 (13.78%), characterized by moderate baseline symptoms (EPDS score of approximately 14 at 13 weeks of gestation), with symptoms first worsening and then improving from late pregnancy through the postpartum period (moderate-increasing) (**Figure 3A**).

**Figure 3:**
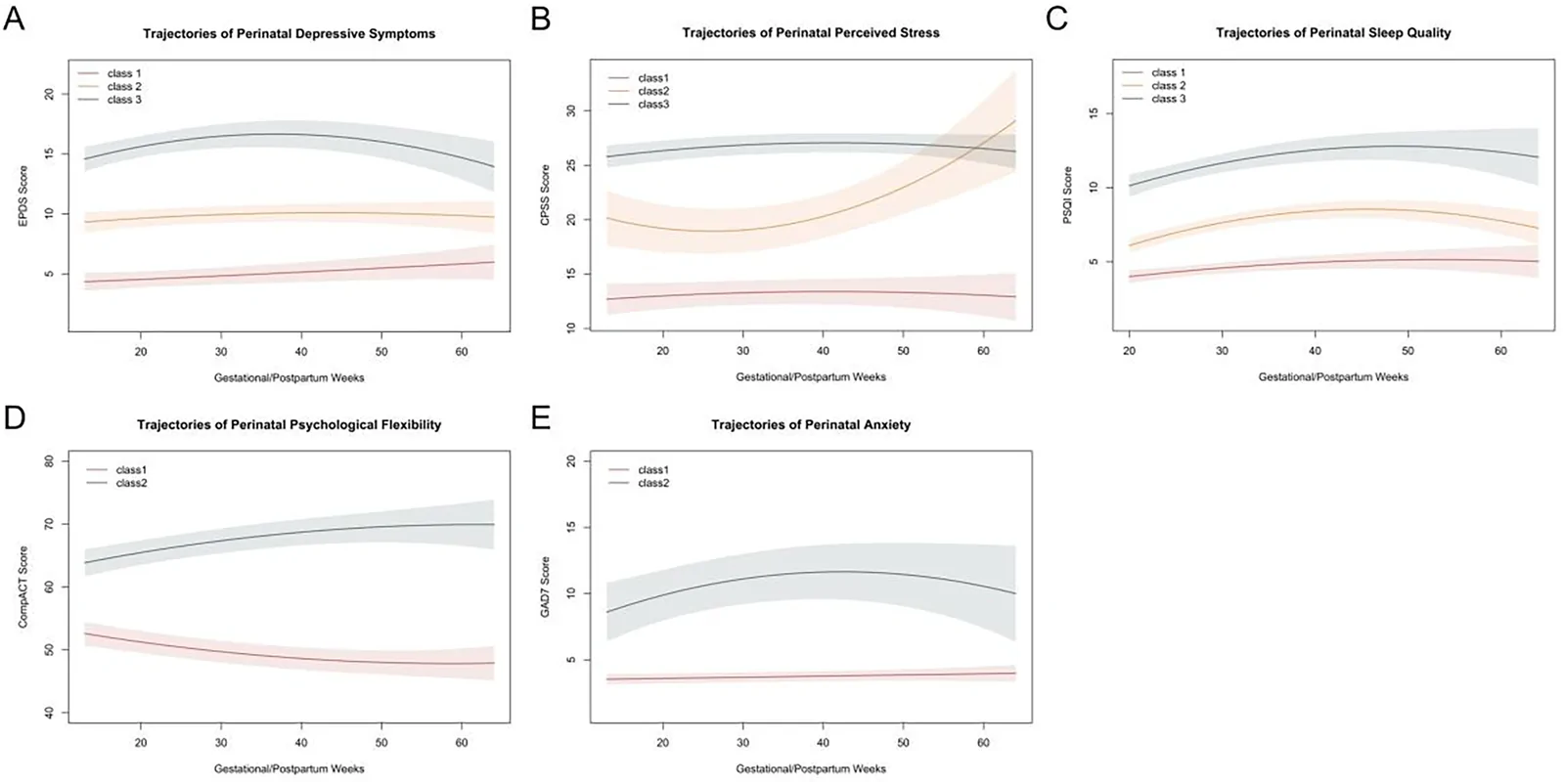
Best-fitting trajectory groups for perinatal **(A)** depressive symptoms (EPDS), **(B)** perceived stress (CPSS), **(C)** sleep quality (PSQI), **(D)** psychological flexibility (CompACT), and **(E)** anxiety (GAD‑7) scores, as identified by group‑based trajectory modeling.

### 3.3 Trajectories of perceived stress (CPSS)

**Supplementary Table S4** presents the fit statistics for the CPSS trajectory models in classes 1 to 6; the three models are the optimal solutions. This approach achieves a balance among fit (AIC = 8,867.63, BIC = 8,920.69), entropy (0.70), avePP (0.83–0.92), and class size (all >5%). These three trajectories are: Class 1 (31.89%), characterized by persistently low levels of psychological stress (low-stable); Class 2 (16.54%), characterized by an increasing trend in stress (moderate-increasing); and Class 3 (51.57%), characterized by persistently high levels (high-stable) (**Figure 3B**).

### 3.4 Trajectories of sleep quality (PSQI)

**Supplementary Table S5** presents the fit statistics for the PSQI trajectory models in classes 1 to 6; the three models are the optimal solutions. This approach achieves a balance among fit (AIC = 5801.44, BIC = 5854.50), entropy (0.75), avePP (0.87–0.90), and class size (all >5%). Class 1 (50.00%) maintained good sleep quality throughout the entire pregnancy (PSQI < 5; ‘good-stable’); Class 2 (38.58%) exhibited mild sleep difficulties that gradually worsened during pregnancy and persisted into the postpartum period (PSQI < 8; ‘mild-worsening’); Class 3 (11.42%) exhibited persistent poor sleep quality throughout pregnancy (PSQI ≥ 8) (‘persistent poor’) (**Figure 3C**).

### 3.5 Trajectories of psychological flexibility (CompACT)

Two trajectories of psychological flexibility emerged (**Supplementary Table S6**). The model achieved a balance between fit (AIC = 10,676.67, BIC = 10,715.58), entropy (0.70), avePP (0.89, 0.93), and class size (all > 5%). Class 1 (57.87%) exhibited low baseline flexibility (CompACT < 55), with a trend of continuous decline, indicating a persistently worsening pattern of psychological flexibility (‘continuously declining’). Class 2 (42.13%) maintained high psychological flexibility throughout the perinatal period (CompACT > 60, ‘high-stable’) (**Figure 3D**).

### 3.6 Trajectories of anxiety symptoms (GAD-7)

Two anxiety trajectories were identified (**Supplementary Table S7**). The model achieved a balance between fit (AIC = 6357.32, BIC = 6436.23), entropy (0.90), avePP (0.89, 0.98), and class size (all > 5%). Class 1 (93.70%) remained at a low and stable level at all time points (GAD-7 score < 5 points), representing stable mild anxiety. Class 2 (6.30%) followed an inverted U-shaped course: symptoms rose from moderate baseline levels to a peak in the third trimester, then declined after delivery, but remained at a moderate level throughout (moderate anxiety) (**Figure 3E**).

### 3.7 Associations of obstetric history with trajectory membership

#### 3.7.1 Depressive symptoms

In a multinomial logistic regression analysis using the low-stable class as the reference (**Table 2**), women with a history of one previous abortion had a significantly higher odds of membership in the mild-stable class (OR = 1.99, 95% CI: 1.09–3.66, P = 0.026), whereas the OR for assignment to the moderate-increasing class was not statistically significant (P = 0.055). Women with a history of ≥ 2 abortions had a significantly higher odds of membership in the mild-stable class (OR = 5.14, 95% CI: 1.79–14.78, P = 0.002) and a significantly higher odds for being classified into the moderate-increasing class (OR = 19.12, 95% CI: 5.26–69.44, P < 0.001). The latter association was also significant in the unadjusted model. Furthermore, the association between the two became stronger as the number of previous abortions increased. There was no statistically significant association between the number of deliveries and the trajectories of any type of depressive symptoms.

**Table 2:** Multinomial logistic regression models of the association between the number of abortions and deliveries with mild-stable and moderate-increasing depression, relative to low-stable depressive symptoms.

Model ^a^	Group	Mild-stable depression		Moderate-increasing depression	
		ORs (95% CI)	*P* value	ORs (95% CI)	*P* value
Number of abortions					
Model 1	0	1.00 (Ref.)		1.00 (Ref.)	
	1	2.04 (1.13, 3.70)	0.018	3.14 (1.19, 8.30)	0.020
	≥ 2	5.23 (1.85, 14.80)	0.002	20.53 (5.97, 70.67)	<0.001
Model 2	0	1.00 (Ref.)		1.00 (Ref.)	
	1	1.99 (1.09, 3.66)	0.026	2.68 (0.98, 7.35)	0.055
	≥ 2	5.14 (1.79, 14.78)	0.002	19.12 (5.26, 69.44)	<0.001
Number of deliveries					
Model 1	0	1.00 (Ref.)		1.00 (Ref.)	
	1	1.07 (0.61, 1.89)	0.810	1.28 (0.55, 2.96)	0.568
	≥ 2	1.25 (0.50, 3.16)	0.635	2.31 (0.71, 7.54)	0.165
Model 2	0	1.00 (Ref.)		1.00 (Ref.)	
	1	0.87 (0.45, 1.66)	0.665	0.80 (0.30, 2.17)	0.943
	≥ 2	1.04 (0.38, 2.81)	0.663	1.47 (0.38, 5.74)	0.581

^a^ Model 1 did not adjust for covariates; Model 2 adjusted for age, education level, average monthly household income, residence, occupation and family history of depression.

#### 3.7.2 Perceived stress

In a multivariate-adjusted multinomial logistic regression analysis using the low-stable psychological stress group as the reference (**Table 3**), women with a history of one previous abortion had a significantly higher odds of membership in the moderate-increasing class (OR = 2.54, 95% CI: 1.06–6.06, P = 0.035), while the odds were significantly higher for those classified in the high-stable class (OR = 2.16, 95% CI: 1.09–4.27, P = 0.027). These findings were also statistically significant in the unadjusted model. However, for women with a history of ≥ 2 previous abortions, no statistically significant association was observed with any classes of psychological stress. There was no statistically significant association between the number of deliveries and the trajectory of any type of psychological stress; however, the OR estimates were all less than 1 after adjusting for covariates.

**Table 3:** Multinomial logistic regression models of the association between the number of abortions and deliveries with moderate-increasing and high-stable stress, relative to low-stable psychological stress.

Model ^a^	Group	Moderate-increasing stress		High-stable stress	
		ORs (95% CI)	*P* value	ORs (95% CI)	*P* value
Number of abortions					
Model 1	0	1.00 (Ref.)		1.00 (Ref.)	
	1	2.73 (1.18, 6.33)	0.019	2.31 (1.21, 4.42)	0.011
	≥ 2	1.73 (0.55, 5.45)	0.347	2.15 (0.94, 4.92)	0.069
Model 2	0	1.00 (Ref.)		1.00 (Ref.)	
	1	2.54 (1.06, 6.06)	0.035	2.16 (1.09, 4.27)	0.027
	≥ 2	1.51 (0.46, 4.95)	0.501	1.92 (0.80, 4.58)	0.142
Number of deliveries					
Model 1	0	1.00 (Ref.)		1.00 (Ref.)	
	1	0.98 (0.43, 2.21)	0.958	1.55 (0.86, 2.82)	0.148
	≥ 2	1.22 (0.36, 4.16)	0.748	1.43 (0.56, 3.67)	0.457
Model 2	0	1.00 (Ref.)		1.00 (Ref.)	
	1	0.89 (0.32, 2.17)	0.718	0.84 (0.81, 3.36)	0.169
	≥ 2	0.69 (0.31, 4.64)	0.792	0.90 (0.56, 4.57)	0.385

^a^ Model 1 did not adjust for covariates; Model 2 adjusted for age, education level, average monthly household income, residence, occupation and family history of depression.

#### 3.7.3 Sleep quality

Number of previous abortions was significantly associated with the mild-worsening sleep trajectory (**Table 4**): one abortion (OR = 2.03, 95% CI: 1.11–3.72, P = 0.022) and two or more abortions (OR = 3.06, 95% CI: 1.32–7.09, P = 0.009); compared with women with no history of miscarriage, the odds of experiencing a decline in sleep quality were increased in both groups. A significant association was also found with a persistently poor sleep trajectory: two or more abortions (OR = 5.51, 95% CI: 1.73–17.59, P = 0.004), whereas one abortion showed no statistically significant association. Number of deliveries was not significantly associated with sleep trajectories.

**Table 4:** Multinomial logistic regression models of the association between the number of abortions and deliveries with mild-worsening and persistent poor sleep quality, relative to good-stable group.

Model ^a^	Group	Mild-worsening sleep quality		Persistent poor sleep quality	
		ORs (95% CI)	*P* value	ORs (95% CI)	*P* value
Number of abortions					
Model 1	0	1.00 (Ref.)		1.00 (Ref.)	
	1	2.05 (1.14, 3.71)	0.017	2.11 (0.81, 5.50)	0.128
	≥ 2	3.09 (1.36, 6.99)	0.007	5.85 (1.97, 17.34)	0.001
Model 2	0	1.00 (Ref.)		1.00 (Ref.)	
	1	2.03 (1.11, 3.72)	0.022	2.04 (0.73, 5.63)	0.170
	≥ 2	3.06 (1.32, 7.09)	0.009	5.51 (1.73, 17.59)	0.004
Number of deliveries					
Model 1	0	1.00 (Ref.)		1.00 (Ref.)	
	1	1.00 (0.57, 1.76)	0.992	1.01 (0.42, 2.42)	0.982
	≥ 2	0.75 (0.30, 1.87)	0.545	1.20 (0.35, 4.17)	0.774
Model 2	0	1.00 (Ref.)		1.00 (Ref.)	
	1	0.89 (0.46, 1.69)	0.713	0.84 (0.30, 2.35)	0.738
	≥ 2	0.69 (0.26, 1.84)	0.464	0.90 (0.21, 3.88)	0.883

^a^ Model 1 did not adjust for covariates; Model 2 adjusted for age, education level, average monthly household income, residence, occupation and family history of depression.

#### 3.7.4 Psychological flexibility

Two trajectories of psychological flexibility were identified: a high-stable and continuously declining groups. Results from a binary logistic model adjusted for covariates revealed a positive association between the number of abortions and a continuously declining psychological flexibility (**Table 5**). Compared with women with no history of abortion, those who had one abortion (OR = 2.44, 95% CI: 1.35–4.49, P = 0.004) and ≥ 2 abortions (OR = 2.61, 95% CI: 1.20–5.99, P = 0.019) had a significantly higher odds of continuously declining class. Similarly, there was no statistically significant association between the number of deliveries and psychological flexibility.

**Table 5:** Binary logistic regression models of the association between the number of abortions and deliveries with continuously declining psychological flexibility and moderate anxiety, relative to high-stable psychological flexibility group and stable mild anxiety group.

Model ^a^	Group	Continuously declining psychological flexibility		Moderate anxiety	
		ORs (95% CI)	*P* value	ORs (95% CI)	*P* value
Number of abortions					
Model 1	0	1.00 (Ref.)		1.00 (Ref.)	
	1	2.48 (1.41, 4.43)	0.002	5.48 (1.62, 25.02)	0.012
	≥ 2	2.64 (1.26, 5.83)	0.012	3.38 (0.60, 18.93)	0.146
Model 2	0	1.00 (Ref.)		1.00 (Ref.)	
	1	2.44 (1.35, 4.49)	0.004	4.18 (1.23, 20.95)	0.034
	≥ 2	2.61 (1.20, 5.99)	0.019	2.14 (0.36, 12.60)	0.383
Number of deliveries					
Model 1	0	1.00 (Ref.)		1.00 (Ref.)	
	1	1.20 (0.71, 2.05)	0.500	2.02 (0.70, 6.21)	0.198
	≥ 2	1.47 (0.64, 3.53)	0.377	0.75 (0.04, 4.62)	0.791
Model 2	0	1.00 (Ref.)		1.00 (Ref.)	
	1	1.15 (0.62, 2.15)	0.667	0.97 (0.28, 3.49)	0.960
	≥ 2	1.51 (0.60, 3.95)	0.392	0.26 (0.01, 2.14)	0.273

^a^ Model 1 did not adjust for covariates; Model 2 adjusted for age, education level, average monthly household income, residence, occupation and family history of depression.

#### 3.7.5 Anxiety

After adjusting for covariates in the binary logistic model, the results showed a positive association between the number of abortions and anxiety (**Table 5**). Compared with women with no history of abortion, women with one abortion had a significantly higher likelihood of moderate anxiety (OR = 4.18, 95% CI: 1.23–20.95, P = 0.034). However, the association between ≥ 2 abortions and moderate anxiety was not statistically significant (OR = 2.14, 95% CI: 0.36–12.60, P = 0.383). Similarly, there was no statistically significant association between the number of deliveries and anxiety during pregnancy.

## 4 Discussion

To our knowledge, this is the first study to simultaneously characterize the longitudinal trajectories of five interrelated psychological domains: depressive symptoms, anxiety, perceived stress, sleep quality, and psychological flexibility, across the full perinatal continuum from 13 weeks of gestation to 24 weeks postpartum, and to link baseline reproductive history to these trajectories in a Chinese clinical sample. This study found significant heterogeneity across all five psychological domains, with two to three distinct developmental trajectories identified for each psychological dimension, confirming that overall average trends mask clinically significant subgroups. There was a significant dose-dependent association between the number of previous abortions and adverse trajectories in depressive symptoms, sleep quality, and psychological flexibility. Women who had two or more previous abortions were more likely to experience severe depression, poor sleep quality, and reduced psychological flexibility. In addition, having had one abortion was also associated with increased perceived stress and anxiety during pregnancy. These associations persisted after adjusting for covariates, whereas the number of previous deliveries was not significantly associated with any of the trajectories.

This study identified three trajectories of depression: low-stable (38.98%), mild-stable (47.24%), and moderate-increasing (13.78%), which are similar to the patterns reported in cohort studies from Canada, Norway, and China (8–11, 33). These studies all found that within populations dominated by low-symptom groups, there is a smaller high-risk subgroup. Notably, Yu et al. found that more than half of the women who screened positive for perinatal depression first did so in early pregnancy (10), which aligns with our observation that the high-risk class already showed moderate symptoms at 13 weeks of gestation. This convergence reinforces calls for depression screening to begin in the first trimester rather than being deferred to the postpartum period.

This study also found a dose-dependent association between the number of previous abortions and adverse psychological trajectories. Previous cross-sectional and cohort studies have confirmed that a history of abortion is associated with increased anxiety, depressive symptoms, and perceived stress in subsequent deliveries (15–17). The Screening for Pregnancy Endpoints (SCOPE) cohort study indicated that women with a history of two previous abortions had a higher risk of depression than those with a history of only one abortion (16). A study of Chinese women with recurrent pregnancy loss also reported that the greater the number of abortions, the more severe the depression and sleep difficulties (20). The findings of this study suggest that cumulative pregnancy loss not only predicts symptom exacerbation at a specific point in time but also indicates a trajectory of persistent worsening or persistent psychological distress throughout the perinatal period (from 13 weeks of gestation to 24 weeks postpartum). This suggests that the psychological sequelae of recurrent abortions are not transient adaptive responses but rather constitute a persistent vulnerability that shapes the entire process of perinatal adaptation. Additionally, women with a history of one or more abortions were significantly more likely to exhibit a sustained decline in psychological flexibility, suggesting that prior pregnancy and pregnancy losses may impair the ability to accept difficult internal experiences and act in accordance with personal values, thereby contributing to perinatal psychological distress (29, 34). This finding suggests that recurrent pregnancy loss may foster experiential avoidance and lead to cognitive fusion due to fear of recurrence, thereby progressively narrowing behavioral patterns throughout pregnancy (34).

The results of this study found that women who had experienced two or more abortions were more likely to experience persistent poor sleep quality during the perinatal period. This is consistent with evidence from previous studies indicating that poor sleep quality in late pregnancy predicts persistent postpartum sleep problems (5), as well as the significant association between sleep disorders and mood disorders in the perinatal population (12, 35). Furthermore, with regard to perceived stress, having only one abortion was significantly associated with the moderate-increasing and high-stable stress trajectories, whereas a higher number of abortions was not statistically associated with perceived stress. This result may be due to the extremely small sample size, which limited statistical power in the subgroup with ≥ 2 abortions, or it may reflect the development of certain adaptive habits or proactive coping mechanisms among women who have experienced multiple abortions (16, 19). For anxiety, one previous abortion was associated with more than four-fold higher odds of following the moderate (inverted U-shaped) trajectory, whereas the estimate for two or more abortions was imprecise owing to the small size of this class (6.30%). The peak of anxiety in the third trimester observed in this class is consistent with the escalation of fear of childbirth and concerns about fetal well-being as delivery approaches (15, 17), and its postpartum decline suggests at least partial symptom resolution once a live birth is achieved. The null findings for number of deliveries across all five domains are compatible with the mixed parity literature. Primiparity has been linked to higher perinatal anxiety and depression in some studies (21), whereas multiparity has been associated with steeper postpartum symptom increases in others (22). This fundings indicate that cumulative loss history carries greater prognostic weight than the number of deliveries itself in this population.

Our findings support three concrete clinical actions. First, the number of previous abortions is inexpensive, routinely collected information that can be used at the first prenatal visit to flag women at elevated risk for worsening depression, deteriorating sleep, declining psychological flexibility, and persistent stress. Given the dose-response gradient observed, women with two or more prior abortions warrant particular attention. Second, because the high-risk depressive symptom class already presented moderate symptoms at 13 weeks of gestation, psychological screening for this subgroup should begin in the first trimester and be repeated across pregnancy and into the postpartum period, rather than relying on a single assessment (7, 10). Third, the identification of declining psychological flexibility as a loss-related vulnerability points to a modifiable intervention target. Acceptance and Commitment Therapy, which directly targets psychological flexibility, has demonstrated feasibility and preliminary efficacy in perinatal populations (6, 23), and may be especially suitable for women with repeated pregnancy loss. The persistence of poor sleep and elevated stress well into the postpartum period further suggests that support should extend beyond delivery and incorporate sleep-focused and stress-management components (5, 12).

Strengths of this study include: a prospective study design with eight repeated measurements spanning from early pregnancy to the postpartum period; simultaneous modeling of five psychological domains within a unified trajectory framework; and the use of validated Chinese scales. Nevertheless, several limitations of this study should be acknowledged. First, the single-center design and sample size limited the precision of estimates for small trajectory classes (e.g., the moderate anxiety class at 6.30%), and substantial sample loss at the end of the postpartum follow-up is reflected in the wide confidence intervals. Therefore, larger-scale, multicenter studies are needed to confirm the association between miscarriage and various psychological dimensions. Second, psychological outcomes relied on self-reports rather than clinical diagnostic interviews. Third, the study did not distinguish between spontaneous and induced abortions, which may have different psychological consequences. Fourth, despite adjustments for covariates, residual confounding due to unmeasured factors cannot be ruled out. Fifth, follow-up ended at 24 weeks postpartum, and trajectories may evolve further during the first postpartum year (12). Finally, although the average posterior probabilities for all retained classes exceeded the recommended threshold of 0.70, the entropy values for some models were moderate (0.70–0.75), suggesting that there may be some uncertainty in classification. Therefore, future research should consider study designs involving large samples and long-term follow-up, as well as validating the trajectories of each psychological dimension in independent samples.

## 5 Conclusions

Perinatal psychological adjustment varies across five domains. Recurrent abortions serve as a strong, dose-dependent predictor of worsening depressive symptoms, decreased sleep quality, and reduced psychological flexibility in pregnant women from early pregnancy through the early postpartum period. Routine assessment of abortion history at the first prenatal visit is a practical and low-cost strategy that can help identify, at an early stage, pregnant women who may benefit from enhanced psychological monitoring and targeted interventions during the perinatal period.

## Data Availability

The raw data supporting the conclusions of this article will be made available by the authors, without undue reservation.
