# Supplementary material for: Trajectories of perinatal depression, anxiety, perceived stress, sleep quality, and psychological flexibility from pregnancy to six months postpartum and their relationship with the number of abortions and deliveries
Source: Front Psychiatry. 2026 Oct 28;17:1973469. doi: 10.3389/fpsyt.2026.1973469 (PMC13752045; doi:10.3389/fpsyt.2026.1973469)
Supplement: Supplementary file 1 [file Table1.docx]

**Table S1** Comparison of baseline characteristics between participants included in (N = 254) and excluded from (n = 44) the trajectory analysis.

| Characteristics |  | Included (N = 254) | Excluded (n = 44) | P value |
| --- | --- | --- | --- | --- |
| Age, n (%) | < 30 | 129 (50.79) | 23 (52.27) | 0.985 |
|  | ≥ 30 | 125 (49.21) | 21 (47.73) |  |
| Education, n (%) | High school or below | 92 (36.22) | 16 (36.36) | 1.000 |
|  | Bachelor or above | 162 (63.78) | 28 (63.64) |  |
| Average monthly household income, n (%) | < 6,000 | 169 (66.54) | 31 (70.45) | 0.736 |
|  | ≥ 6,000 | 85 (33.46) | 13 (29.55) |  |
| Residence, n (%) | Urban | 171 (67.32) | 35 (79.55) | 0.149 |
|  | Rural | 83 (32.68) | 9 (20.45) |  |
| Occupation, n (%) | Unemployed | 79 (31.10) | 13 (29.55) | 0.976 |
|  | Employed | 175 (68.90) | 31 (70.45) |  |
| Family history of depression, n (%) | No | 240 (94.49) | 41 (93.18) | 0.724 |
|  | Yes | 14 (5.51) | 3 (6.82) |  |
| Mode of conception, n (%) | Natural | 250 (98.43) | 44 (100.00) | 1.000 |
|  | Assisted | 4 (1.57) | 0 (0.00) |  |
| Exercise frequency / week, n (%) | ≥ 5 times | 45 (17.72) | 6 (13.64) | 0.764 |
|  | 3–4 times | 60 (23.62) | 11 (25.00) |  |
|  | 1–2 times | 59 (23.23) | 13 (29.55) |  |
|  | Rarely | 90 (35.43) | 14 (31.82) |  |
| Complications, n (%) | No | 225 (88.58) | 41 (93.18) | 0.596 |
|  | Yes | 29 (11.42) | 3 (6.82) |  |
| Number of previous abortions, n (%) | 0 | 128 (50.39) | 24 (54.55) | 0.517 |
|  | 1 | 86 (33.86) | 16 (36.36) |  |
|  | ≥ 2 | 40 (15.75) | 4 (9.09) |  |
| Number of previous deliveries, n (%) | 0 | 127 (50.00) | 26 (59.09) | 0.187 |
|  | 1 | 99 (38.98) | 11 (25.00) |  |
|  | ≥ 2 | 28 (11.02) | 7 (15.91) |  |
| Parental relationship satisfaction, M (IQR) | | 9.00 (8.00, 10.00) | 9.00 (8.00, 10.00) | 0.912 |
| Marital relationship satisfaction, M (IQR) | | 9.00 (8.00, 10.00) | 9.00 (8.00, 10.00) | 0.609 |
| Mother-in-law relationship satisfaction, M (IQR) | | 8.00 (7.00, 10.00) | 9.00 (7.00, 10.00) | 0.338 |

Notes: Categorical variables are presented as n (%) and were compared using the Chi-square test or Fisher’s exact test (family history of depression, mode of conception, complications); continuous variables are presented as median (IQR) and were compared using the Mann–Whitney U test. Excluded participants were those who completed fewer than three of the seven follow-up assessments.

**Table S2** Cronbach's α coefficients.

| Time points | N | Depression  (EPDS) | Anxiety  (GAD-7) | Perceived stress  (CPSS) | Sleep quality  (PSQI) | Psychological flexibility  (CompACT) | Family function  (APGAR) |
| --- | --- | --- | --- | --- | --- | --- | --- |
| Gestation |  |  |  |  |  |  |  |
| 13 weeks | 298 | 0.850 | 0.883 | 0.799 | NA | 0.852 | NA |
| 20 weeks | 269 | 0.848 | 0.901 | 0.845 | 0.842 | 0.900 | 0.905 |
| 26 weeks | 254 | 0.836 | 0.913 | 0.851 | 0.866 | 0.900 | 0.935 |
| 32 weeks | 243 | 0.848 | 0.916 | 0.877 | 0.898 | 0.908 | 0.929 |
| 37 weeks | 230 | 0.857 | 0.913 | 0.866 | 0.726 | 0.904 | 0.949 |
| Postpartum |  |  |  |  |  |  |  |
| 1 weeks | 139 | 0.863 | 0.931 | 0.878 | 0.717 | 0.904 | 0.942 |
| 6 weeks | 108 | 0.851 | 0.865 | 0.869 | 0.869 | 0.889 | 0.936 |
| 24 weeks | 67 | 0.806 | 0.885 | 0.829 | 0.858 | 0.906 | 0.945 |

**Table S3** Goodness of fit for the group-based trajectory model EPDS.

| k | LL | Entropy | AIC | BIC | %class 1 (avePP)^*^ | %class 2 (avePP)^*^ | %class 3 (avePP)^*^ | %class 4 (avePP)^*^ | %class 5 (avePP)^*^ | %class 6 (avePP)^*^ |
| --- | --- | --- | --- | --- | --- | --- | --- | --- | --- | --- |
| 1 | -3862.34 | 1.00 | 7738.68 | 7763.44 | 100.0% (1.00) |  |  |  |  |  |
| 2 | -3849.46 | 0.67 | 7720.91 | 7759.83 | 81.10% (0.92) | 18.90% (0.83) |  |  |  |  |
| **3** | **-3842.60** | **0.74** | **7715.20** | **7768.26** | **47.24% (0.85)** | **38.98% (0.91)** | **13.78% (0.92)** |  |  |  |
| 4 | -3841.33 | 0.53 | 7720.66 | 7787.87 | 33.07% (0.70) | 1.18% (0.88) | 49.61% (0.70) | 16.14% (0.83) |  |  |
| 5 | -3839.65 | 0.53 | 7725.29 | 7806.65 | 36.22% (0.68) | 42.52% (0.67) | 1.57% (0.74) | 3.15% (0.66) | 16.54% (0.81) |  |
| 6 | -3832.28 | 0.62 | 7718.57 | 7814.07 | 15.75% (0.60) | 24.80% (0.75) | 5.91% (0.73) | 37.01% (0.75) | 3.54% (0.61) | 12.99% (0.91) |

Notes: ^*^%class (avePP) denotes the proportion of members assigned to this class and the average posterior probability. BIC = Bayesian information criterion; AIC: Akaike’ s Information Criterion; LL: log-likelihood; avePP: Average Predicted Probability.

**Table S4** Goodness of fit for the group-based trajectory model CPSS.

| k | LL | Entropy | AIC | BIC | %class 1 (avePP)^*^ | %class 2 (avePP)^*^ | %class 3 (avePP)^*^ | %class 4 (avePP)^*^ | %class 5 (avePP)^*^ | %class 6 (avePP)^*^ |
| --- | --- | --- | --- | --- | --- | --- | --- | --- | --- | --- |
| 1 | -4444.00 | 1.00 | 8902.00 | 8926.76 | 100.0% (1.00) |  |  |  |  |  |
| 2 | -4429.37 | 0.75 | 8880.74 | 8919.65 | 38.98% (0.90) | 61.02% (0.95) |  |  |  |  |
| **3** | **-4418.82** | **0.70** | **8867.63** | **8920.69** | **31.89% (0.87)** | **16.54% (0.68)** | **51.57% (0.92)** |  |  |  |
| 4 | -4412.78 | 0.74 | 8863.55 | 8930.76 | 31.50% (0.88) | 49.61% (0.91) | 16.93% (0.67) | 1.97% (0.78) |  |  |
| 5 | -4411.28 | 0.68 | 8868.56 | 8949.91 | 32.68% (0.86) | 4.72% (0.66) | 0.39% (0.99) | 14.96% (0.64) | 47.24% (0.80) |  |
| 6 | -4395.40 | 0.78 | 8844.80 | 8940.31 | 7.48% (0.83) | 29.92% (0.79) | 3.94% (0.76) | 3.54% (0.72) | 4.33% (0.84) | 50.79% (0.90) |

Notes: ^*^%class (avePP) denotes the proportion of members assigned to this class and the average posterior probability. BIC = Bayesian information criterion; AIC: Akaike’ s Information Criterion; LL: log-likelihood; avePP: Average Predicted Probability.

**Table S5** Goodness of fit for the group-based trajectory model CompACT.

| k | LL | Entropy | AIC | BIC | %class 1 (avePP)^*^ | %class 2 (avePP)^*^ | %class 3 (avePP)^*^ | %class 4 (avePP)^*^ | %class 5 (avePP)^*^ | %class 6 (avePP)^*^ |
| --- | --- | --- | --- | --- | --- | --- | --- | --- | --- | --- |
| 1 | -5340.27 | 1.00 | 10694.55 | 10719.31 | 100.0% (1.00) |  |  |  |  |  |
| **2** | **-5327.34** | **0.70** | **10676.67** | **10715.58** | **57.87% (0.93)** | **42.13% (0.89)** |  |  |  |  |
| 3 | -5323.21 | 0.74 | 10676.42 | 10729.48 | 56.69% (0.89) | 1.18% (0.84) | 42.13% (0.89) |  |  |  |
| 4 | -5308.98 | 0.78 | 10655.95 | 10723.16 | 37.01% (0.94) | 34.25% (0.82) | 20.87% (0.90) | 7.87% (0.79) |  |  |
| 5 | -5305.36 | 0.80 | 10656.73 | 10738.08 | 36.61% (0.92) | 34.25% (0.82) | 0.39% (0.99) | 20.87% (0.91) | 7.87% (0.79) |  |
| 6 | -5304.22 | 0.70 | 10662.43 | 10757.94 | 22.44% (0.63) | 13.78% (0.66) | 0.39% (0.99) | 34.25% (0.81) | 20.87% (0.90) | 8.27% (0.80) |

Notes: ^*^%class (avePP) denotes the proportion of members assigned to this class and the average posterior probability. BIC = Bayesian information criterion; AIC: Akaike’ s Information Criterion; LL: log-likelihood; avePP: Average Predicted Probability.

**Table S6** Goodness of fit for the group-based trajectory model GAD7.

| k | LL | Entropy | AIC | BIC | %class 1 (avePP)^*^ | %class 2 (avePP)^*^ | %class 3 (avePP)^*^ | %class 4 (avePP)^*^ | %class 5 (avePP)^*^ | %class 6 (avePP)^*^ |
| --- | --- | --- | --- | --- | --- | --- | --- | --- | --- | --- |
| 1 | -3210.66 | 1.00 | 6435.31 | 6460.07 | 100.0% (1.00) |  |  |  |  |  |
| **2** | **-3182.66** | **0.90** | **6357.32** | **6436.23** | **93.70% (0.98)** | **6.30% (0.89)** |  |  |  |  |
| 3 | -3178.85 | 0.79 | 6387.70 | 6440.76 | 42.91% (0.90) | 50.00% (0.90) | 7.09% (0.92) |  |  |  |
| 4 | -3175.72 | 0.73 | 6389.45 | 6456.66 | 46.06% (0.87) | 2.36% (0.71) | 44.49% (0.83) | 7.09% (0.96) |  |  |
| 5 | -3172.86 | 0.65 | 6391.71 | 6473.07 | 11.42% (0.57) | 38.19% (0.82) | 40.94% (0.78) | 2.36% (0.71) | 7.09% (0.96) |  |
| 6 | -3161.35 | 0.70 | 6376.71 | 6472.22 | 29.13% (0.71) | 3.54% (0.92) | 2.36% (0.76) | 40.55% (0.82) | 21.26% (0.78) | 3.15% (0.97) |

Notes: ^*^%class (avePP) denotes the proportion of members assigned to this class and the average posterior probability. BIC = Bayesian information criterion; AIC: Akaike’ s Information Criterion; LL: log-likelihood; avePP: Average Predicted Probability.

**Table S7** Goodness of fit for the group-based trajectory model PSQI.

| k | LL | Entropy | AIC | BIC | %class 1 (avePP)^*^ | %class 2 (avePP)^*^ | %class 3 (avePP)^*^ | %class 4 (avePP)^*^ | %class 5 (avePP)^*^ | %class 6 (avePP)^*^ |
| --- | --- | --- | --- | --- | --- | --- | --- | --- | --- | --- |
| 1 | -2911.54 | 1.00 | 5837.09 | 5861.85 | 100.0% (1.00) |  |  |  |  |  |
| 2 | -2894.71 | 0.73 | 5811.41 | 5850.32 | 80.71% (0.94) | 19.29% (0.85) |  |  |  |  |
| **3** | -2885.72 | 0.75 | 5801.44 | 5854.50 | 38.58% (0.87) | 50.00% (0.89) | 11.42% (0.91) |  |  |  |
| 4 | -2885.72 | 0.61 | 5809.44 | 5876.65 | 40.16% (0.85) | 0.00% (NA) | 48.43% (0.69) | 11.42% (0.90) |  |  |
| 5 | -2882.36 | 0.66 | 5810.73 | 5892.09 | 23.23% (0.61) | 25.98% (0.75) | 11.02% (0.82) | 31.50% (0.81) | 8.27% (0.92) |  |
| 6 | -2878.35 | 0.60 | 5810.69 | 5906.20 | 9.45% (0.69) | 0.00% (NA) | 40.16% (0.73) | 32.28% (0.64) | 6.69% (0.67) | 11.42% (0.93) |

Notes: ^*^%class (avePP) denotes the proportion of members assigned to this class and the average posterior probability. BIC = Bayesian information criterion; AIC: Akaike’ s Information Criterion; LL: log-likelihood; avePP: Average Predicted Probability.
